# Structural insight into YcbB-mediated beta-lactam resistance in *Escherichia coli*

**DOI:** 10.1038/s41467-019-09507-0

**Published:** 2019-04-23

**Authors:** Nathanael A. Caveney, Guillermo Caballero, Henri Voedts, Ana Niciforovic, Liam J. Worrall, Marija Vuckovic, Matthieu Fonvielle, Jean-Emmanuel Hugonnet, Michel Arthur, Natalie C. J. Strynadka

**Affiliations:** 1 Department of Biochemistry and Molecular Biology and the Centre for Blood Research, University of British Columbia, Vancouver, V6T 1Z3 BC Canada; 2 Sorbonne Université, Sorbonne Paris Cité, Université Paris Descartes, Université Paris Diderot, INSERM, Centre de Recherche des Cordeliers, CRC, 75006 Paris, France

**Keywords:** X-ray crystallography, Antibiotics

## Abstract

The bacterial cell wall plays a crucial role in viability and is an important drug target. In *Escherichia coli*, the peptidoglycan crosslinking reaction to form the cell wall is primarily carried out by penicillin-binding proteins that catalyse D,D-transpeptidase activity. However, an alternate crosslinking mechanism involving the L,D-transpeptidase YcbB can lead to bypass of D,D-transpeptidation and beta-lactam resistance. Here, we show that the crystallographic structure of YcbB consists of a conserved L,D-transpeptidase catalytic domain decorated with a subdomain on the dynamic substrate capping loop, peptidoglycan-binding and large scaffolding domains. Meropenem acylation of YcbB gives insight into the mode of inhibition by carbapenems, the singular antibiotic class with significant activity against L,D-transpeptidases. We also report the structure of PBP5-meropenem to compare interactions mediating inhibition. Additionally, we probe the interaction network of this pathway and assay beta-lactam resistance in vivo. Our results provide structural insights into the mechanism of action and the inhibition of L,D-transpeptidation, and into YcbB-mediated antibiotic resistance.

## Abstract (web summary)

In *E. coli*, alternate peptidoglycan crosslinking reactions carried out by the L,D-transpeptidase YcbB can lead to beta-lactam resistance. Here, Caveney et al. solve the crystal structure of YcbB and shed light into its mechanism of action and into YcbB-mediated antibiotic resistance.

## Introduction

The bacterial cell wall plays a critical role in the strength and viability of bacteria within their natural environs. Underscored by the development of beta-lactam antibiotics such as penicillins and cephalosporins, biosynthesis of the major structural component of the bacterial cell wall, peptidoglycan (PG), has been long recognized as an important drug target. PG is composed of extended polymerized glycan strands of alternating beta-1,4 linked *N*-acetyl-glucosamine-*N*-acetyl-muramic acid which crosslink into a net-like mesh via short peptide segments covalently attached at the C3–OH position of the latter sugar^1^. The precursor subunits of PG are synthesised in the cytosol and transferred to an undecaprenyl phosphate lipid carrier^2^. This moiety is then flipped to the periplasmic face of the membrane, where polymerisation into glycan strands, release from lipid carrier and peptide crosslinking to the existing PG sacculus occur in concert^2,3^.

In *Escherichia coli*, this peptide crosslinking reaction is primarily carried out by class A and B penicillin-binding proteins (PBPs). These proteins have D,D-transpeptidase activity and catalyse the cleavage of a D-Ala^4^-D-Ala^5^ peptide bond of the acyl donor and subsequent crosslinking of the D-Ala^4^ carbonyl to the primary amine of a diaminopimelic acid (DAP) residue on the acceptor. This results in a D-Ala^4^_donor_-DAP^3^_acceptor_ crosslink^1^. This D,D-transpeptidase activity can be blocked by beta-lactam antibiotics, which act as a substrate mimetic of the donor strand peptide bond. Inhibition of this final and crucial step in PG biosynthesis results in destabilisation of the cell wall and ultimately cell death^4^.

Despite the success of beta-lactam antibiotics in the clinic, it has recently been shown that an alternate crosslinking mechanism can lead to the bypass of PBP mediated D,D-transpeptidation. The L,D-transpeptidase YcbB (alternately named LdtD), with remarkably few additional accessory factors, is able to undertake this recovery of PG crosslinking function, even in the presence of many clinically used beta-lactam antibiotics. The factors required include the upregulation of alarmone (ppGpp) synthesis, the class C monofunctional PBP5, and an associated glycosyltransferase (GTase) activity attributed to the class A bifunctional PBP1b^5^ which harbours both GTase and TPase activities in distinct active sites. As with D,D-crosslinked PG, the bypass pathway initiates with GTase catalyzed polymerisation of PG; the resultant growing product strand is acted upon by PBP5 which catalyses a carboxypeptidase reaction targeting removal of the terminal D-Ala^5^ resulting in a donor glycan-tetrapeptide substrate for YcbB. In the last step of the bypass pathway, YcbB catalyses L,D-transpeptidation using this tetrapeptide-containing donor to form a DAP^3^_donor_-DAP^3^_acceptor_ crosslink^5^. L,D-transpeptidases, including YcbB, are not efficaciously inactivated by beta-lactams^5^. The formation of a thioester-containing L,D-transpeptidase-beta-lactam adduct is slow for penicillins and cephalosporins^6^. The acylation reaction is followed by hydrolysis, thereby preventing full inactivation of the enzyme^6^. The D,D-carboxypeptidase PBP5, is inhibited only at high concentrations of beta-lactams^7^.

Although YcbB is resistant to most beta-lactam antibiotics, it is susceptible to inhibition by carbapenem antibiotics such as meropenem and imipenem^5^. Carbapenem antibiotics are a subset of beta-lactam antibiotics which have previously been shown to inhibit other L,D-transpeptidases^8^. This class of antibiotic is of interest, not only due to its ability to inhibit YcbB, but as well the carboxypeptidase activity of PBP5 and the D,D-transpeptidase activity of PBP1b. Further insight into the inhibition of the enzymes in the D,D-transpeptidase bypass pathway could lead to the development of novel inhibitors to better address the current beta-lactam resistance crisis. Additionally, it was recently shown that copper can inhibit L,D-transpeptidation of bacterial cell walls and specifically inhibit YcbB^9^. This plays an important role in the inherent antimicrobial activity of copper.

In this work, we report the structure of *E. coli* YcbB acylated with meropenem. YcbB is seen to consist of a conserved L,D-transpeptidase catalytic domain, with the notable additions of a subdomain on the substrate capping loop, a PG binding domain, and a large scaffolding domain potentially important in mediating partner interactions. This complex domain architecture is unique in comparison to that of well characterised Gram positive and *Mycobacterium* L,D-transpeptidases. In addition, we report in parallel, the structure of a PBP5-meropenem acyl enzyme complex in order to completely describe the molecular interactions which facilitate meropenem inhibition of the YcbB and PBP5 partners. Our results provide structural insight into D,D-transpeptidase bypass in *E. coli* and structural insight into the role of L,D-transpeptidation of PG in Gram negative bacteria. Additionally, we probe the protein interaction network and affinities for the D,D-transpeptidase bypass pathway and assay YcbB mediated beta-lactam resistance in an in vivo setting allowing further mechanistic insight into this drug resistance pathway and generated phenotype.

## Results and discussion

### *E. coli* YcbB X-ray crystallographic structure

**Fig. 1:**
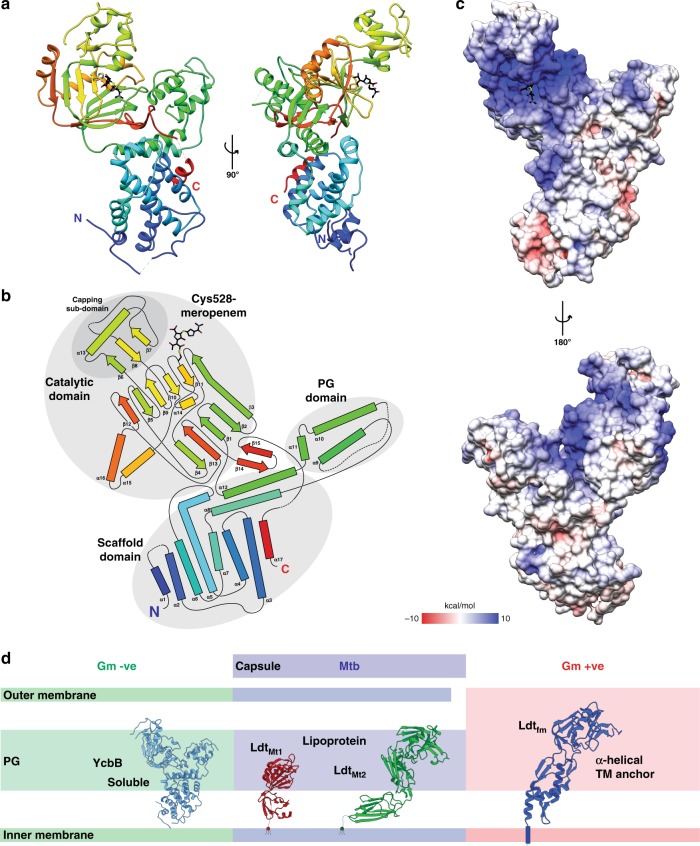
Overall architecture of *E. coli* YcbB. **a** The crystal structure of YcbB-meropenem acyl-enzyme complex in ribbon representation, coloured in rainbow from N-terminus (blue) to C-terminus (red). Meropenem stick representation is coloured in black and by heteroatom. Two views related by a 90° rotation along the *y*-axis. **b** Topology diagram of YcbB-meropenem acyl-enzyme complex, coloured as in **a**. The catalytic, PG and scaffolding domains are annotated and circled in grey. **c** Electrostatic surface representation of YcbB in two views related by a 180° rotation around the *y*-axis. **d** Diversity of structurally characterised L,D-transpeptidases. Gram negative (green background), soluble YcbB in sky blue; Mycobacterial (blue background), lipoprotein Ldt_Mt1_ (PDBID 5E5L^8^) and Ldt_Mt2_ (PDBID 5DU7^8^) in red and green; Gram positive (red background), TM anchored Ldt_fm_ (1ZAT^16^) in dark blue

To investigate the role of YcbB in beta-lactam resistance at the atomic level and provide insight into L,D-transpeptidase activity in Gram negative bacteria, the X-ray crystallographic structure of *E. coli* YcbB was pursued. Cocrystals, generated in the presence of 1 mM meropenem at pH 7.5, displayed *P4*_*3*_*2*_*1*_*2* symmetry with unit cell dimensions of *a* = 126.5 Å, *b* = 126.5 Å, *c* = 88.8 Å and a diffraction resolution of 2.76 Å. There is one molecule of YcbB in the asymmetric unit. The structure solution was phased using a distinct SIR dataset of a heavy atom (ethyl mercury phosphate) soaked crystal with final refinement statistics presented in Supplementary Table 1. The resulting maps showed well resolved electron density for the majority of the enzyme chain (Supplementary Fig. 1), allowing near complete tracing of the YcbB model (see Methods). As in solution, the structure indicates a monomeric form of the enzyme with no obvious crystallographic formation of larger oligomers. The overall dimensions of the enzyme are 74 × 73 × 40 Å with a total surface area of ~24,400 Å^2^, and with a largely positive electrostatic distribution across the catalytic domain and largely apolar distribution across the PG and scaffolding domains. An extended electropositive active site demarcated by bound meropenem antibiotic lies across the catalytic domain with appropriate clefts for both donor and acceptor substrates adjacent and continuous (Fig. 1). The observed overall architecture of YcbB (Fig. 1) has several distinct features from all currently deposited L,D-transpeptidase structures, as suggested from the unique insertions and substitutions in sequence alignments with previously characterized Gram positive and mycobacterial species (7.1–14.3% sequence identity; Supplementary Fig. 2). The well ordered central catalytic domain, comprised of residues 375–576, forms a canonical L,D-transpeptidase fold harbouring an extended active site cleft with the expected conserved active site motif^10^ (HX_15–18_[S/T]XGC*h*[R/N], where X represents any residue, and *h* is any hydrophobic residue), but with the notable insertion of a unique substrate capping sub-domain (residues 422–495), unprecedented in size and observed secondary structural elements and lying adjacent to the meropenem-acylated catalytic site. On the opposite face of the central catalytic domain, an also unprecedented N-terminal region, central helix, and C-terminal helical tail (residues 37–233, 352–374, and 605–615 respectively) are proximal and form a collective helical bundle we term the scaffold domain, and predict to play a potential role in protein–protein interactions in YcbB-mediated reistance^5^. Residues 234–351 form a small antiparallel three helical bundle, with features reminiscent of previously observed PG binding regions—and thus we term here the PG domain^11^. Residues 577–604 form a beta-hairpin linker between the catalytic domain and the C-terminal recursion.

### YcbB scaffold domain structure

The scaffold domain of YcbB is comprised of ten helices packed into a helical bundle. The seven core α-helices are contributed by residues 100–213, with the eighth α-helix contributed from the C-terminal recursion involving residues 600–615 (Fig. 1). Two additional helices (residues 214–233 and 352–374) cap the domain and structurally link the catalytic and PG binding domains. Residues downstream (31–99) form a generally less ordered region with interspersed α-helical segments which sit distal to the other domains of YcbB. The scaffold domain is unique to the YcbB sub-family of L,D-transpeptidases, suggesting a specific functional and/or regulatory role we predict to involve interaction with the known YcbB unique protein partners PBP5 and PBP1b^5^. These interactions are likely crucial for localization of YcbB to the site of late stage PG synthesis, as, unlike L,D-transpeptidases from Gram positive bacteria or *Mycobacterium*, YcbB does not have a α-helical transmembrane tether or N-terminal lipidation (Fig. 1d). Rather, YcbB is predicted to be targeted to the periplasmic space using a characteristic sec-dependent signalling sequence (residues 1–30—SignalP 4.1^12^), which is subsequently processed through proteolytic cleavage with release of soluble YcbB.

### YcbB peptidoglycan binding domain

**Fig. 2:**
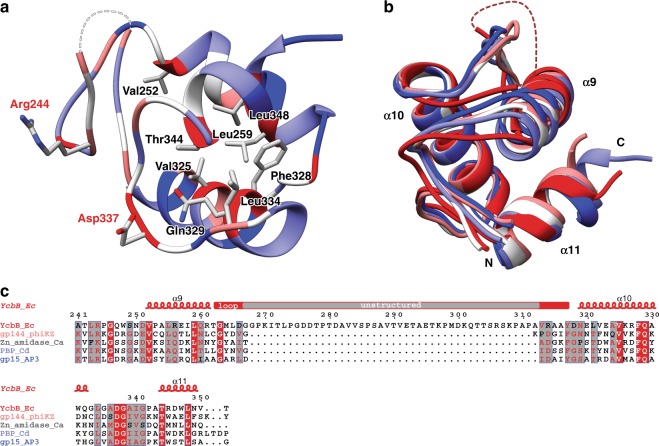
Structure of the *E. coli* YcbB peptidoglycan binding domain. **a** Ribbon representation of the *E. coli* YcbB peptidoglycan binding domain, residues 241–351, with residue conservation mapped to backbone colour from red to blue in decreasing conservation. Conserved core residues labelled in black and key, surface exposed, peptidoglycan binding residues labelled in red. **b** Overlay of YcbB peptidoglycan binding domain and high scoring peptidoglycan binding domains from a structural homology search on the Dali server^14^. YcbB in red, gp144, a *Pseudomonas aeruginosa* phage phiKZ endolysin in salmon (PDB ID 3BKH^15^), a *Clostridium acetobutylicum* zinc-dependent amidase in white (PDB ID 4XXT), a *Clostridium difficile* PBP in pale blue (PDB ID 5TV7), and gp15, a *Burkholderia* AP3 phage endolysin in dark blue (PDB ID 5NM7^11^). Peptidoglycan binding domain structures are coloured from red to blue based on decreasing structural homology. **c** The sequence of *E. coli* YcbB (YcbB_Ec) aligned with peptidoglycan binding domains from phiKZ gp144 (gp144_phiKZ), *C. acetobutylicum* zinc-dependent amidase (Zn_amidase_Ca), *C. difficile* PBP (PBP_Cd), and AP3 gp15 (gp15_AP3). Secondary structure of *E. coli* YcbB is displayed atop the sequence alignment, with the extended loop shown in red and the unstructured region in grey

Many characterized L,D-transpeptidases have been shown to contain PG binding motifs/domains, often a LysM domain consisting of a βααβ fold with the two α-helices located on the same side of an antiparallel beta-sheet^13^. In YcbB, residues 234–351 form a small antiparallel three helical bundle which, based on these prior observations, is suggestive of a putative PG binding domain (Fig. 2) albeit a PG binding domain more characteristic of lytic phage enzymes and a Zn-dependent bacterial amidase than PG binding motifs observed in other structurally characterized L,D-transpeptidases. Structural homology analysis, via the DALI protein structure comparison server^14^, finds close structural homologues—gp144, a *Pseudomonas aeruginosa* phage phiKZ endolysin (PDB ID 3BKH^15^, Z-score 9.7, RMSD 1.9), a *Clostridium acetobutylicum* zinc-dependent amidase (PDB ID 4XXT, Z-score 9.6, RMSD 1.9), a *Clostridioides difficile* PBP (PDB ID 5TV7, Z-score 9.4, RMSD 2.0), and gp15, a *Burkholderia* AP3 phage endolysin (PDB ID 5NM7^11^, Z-score 9.4, RMSD 2.0). The three antiparallel helices are common to all five PG binding domains, though in YcbB the loop region between the first and second helices contains a dynamic and relatively conserved extension (residues 262–318 with the central region therein highly disordered). Due to the lack of this loop extension in the above identified structural homologues, it is possible that these residues become more stabilised upon PG binding, increasing the effective interaction interface or specificity with PG substrates in this family. The PG domain of YcbB contains many conserved residues with other YcbB family members primarily in the hydrophobic core of the motif (Val252, Leu259, Ala324, Val325, Phe328, Gln329, Leu334, Thr344, and Leu348) as well as in two surface exposed residues, Arg244 and Asp337, which have been implicated previously as essential in PG binding^11^.

### YcbB catalytic domain

**Fig. 3:**
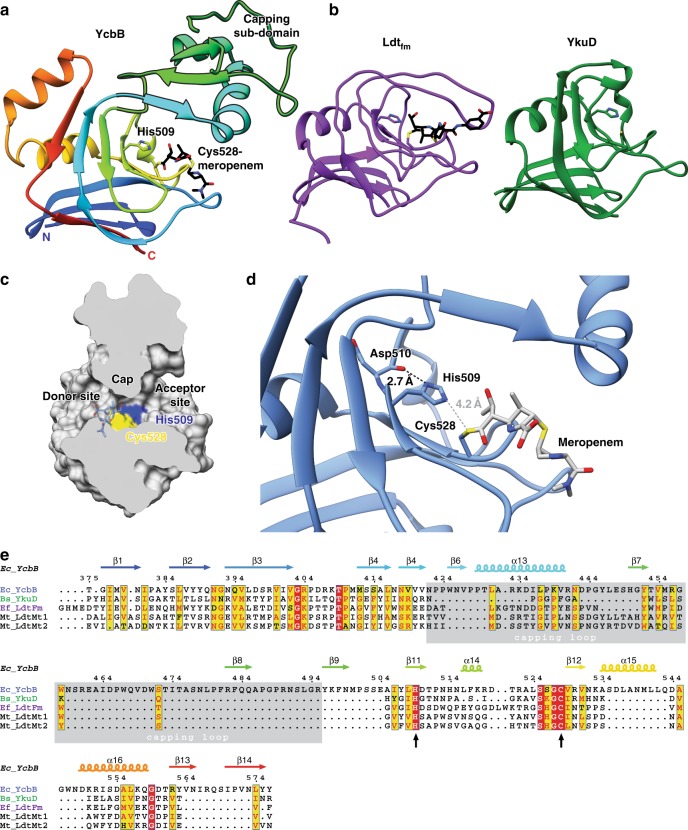
Structure of the *E. coli* YcbB catalytic domain. **a** A ribbon representation of the YcbB catalytic domain, residues 375–576, coloured in rainbow from red (N-terminus) to blue (C-terminus). Meropenem is represented in black and coloured by heteroatom. The unique capping sub-domain is highlighted in a bold silhouette. **b** The catalytic domain architecture of *Enterococcus faecium* Ldt_Fm_ (PDB ID 3ZGP^17^) and *Bacillus subtilis* YkuD (PDB ID 1Y7M^13^) highlighting the differences seen among L,D-transpeptidase catalytic domains in the capping loop region. **c** Cut surface representation of the YcbB catalytic domain, showing the capped cleft formed between the donor and acceptor sites by the capping loop sub-domain. The catalytic Cys528 and His509 are coloured in yellow and blue respectively. Meropenem (blue) is overlaid in its position in the donor site, acylating the catalytic cysteine. **d** The active site architecture of *E. coli* YcbB. The catalytic Cys528 is acylated by meropenem and the sulphide is rotated away from the N^τ^ of His509 (4.2 Å) which activated the cystine prior to acylation. The N^π^ of His509 is seen to be in close proximity with the backbone carbonyl of the adjacent Asp510 (2.7 Å), stabilising and promoting formation of the histidine cation. **e** The sequence of *E. coli* YcbB (Ec_YcbB) aligned with the aforementioned catalytic domains from *E. faecium* and *B. subtilis* (Ef_Ldtfm and Bs_Yku, respectively) as well as Ldt_Mt1_ and Ldt_Mt2_ from *Mycobacterium tuberculosis* (Mt_LdtMt1 and Mt_LdtMt2, respectively)

The central YcbB catalytic domain adopts a characteristic L,D-transpeptidase fold (Fig. 3) constructed of two (five and six stranded) curved, mixed beta-sheets disposed in a clam-shell like manner with α-helices 15 and 16 situated in the cleft between. Acting as a hinge, beta-strand 3 participates in both beta-sheets of the domain. Situated just downstream of beta-strand 11, the active site is demarcated by the meropenem acylated catalytic cysteine nucleophile (Cys528) with the conserved histidine base (His509) protruding from beta-strand 10. The oxyanion hole, presumed to stabilize the developing tetrahedral oxyanion transition state during acylation and deacylation is comprised of the main chain nitrogen atoms of Cys528 and Tyr507.

One notable feature of the catalytic domain of YcbB is the large substrate capping sub-domain (residues inserted between beta-strands 5 and 9). This region has been seen to be variant in currently solved structures of L,D-transpeptidases, ranging from small turns (YkuD^13^) to larger loops of up to 19 residues which extend over the active site (Ldt_fm_^16,17^) (Fig. 3b). However, YcbB is the first structure characterized which contains significant secondary structural elements and consequent folded sub-domain in this region (residues 423–487). In YcbB this putative substrate capping sub-domain is comprised of a small three stranded beta-sheet (β6,8,7) and α-helix 13, which sits perpendicular to the beta-sheet at one end. Between beta-strand 7 and 8 there is a large loop region from residue 453 to 481. Beta-strand 6 and α-helix 13 are seen to hinge towards the active site in the meropenem acyl-enzyme complex structure. It has been proposed previously that the substrate capping loop in earlier structures serves to provide inherent flexibility during roles in substrate entrance, capping, and release^18^. Despite the more structured and significant nature of the YcbB sub-domain, the density, although traceable, was less well resolved and displayed generally higher temperature factors, all in keeping with an analogous inherent flexible nature and potential similar role(s) in catalysis. Further, crystallographic structures and molecular dynamics simulations of Ldt_Mt2_ from *Mycobacterium tuberculosis* have recently shown that the substrate capping loop can have variable flexibility dependent on the presence of differing inhibitors with obvious implications for drug discovery efforts against this family^18,19^. To probe further the potential analogy with the more structured sub-domain in YcbB, we performed 10 ns molecular dynamics simulations (Supplementary Fig. 3) using a CHARMM36 force field (see Methods) which additionally supported the propensity for its highly mobile nature. Interestingly, simulations with meropenem showed increased RMSD spread in the capping loop region but an overall stabilisation of the remainder of the catalytic domain, while in apoenzyme simulations there was increased motion not only in the capping sub-domain but as well over the entirety of the catalytic domain. It is likely that in the action of YcbB on extended native PG substrates, this motion would provide for additional interactions to facilitate binding and product release. In the YcbB-meropenem acyl-enzyme complex structure, the capping subdomain is seen to be stabilised in a position adjacent to the active site, creating a distinct cap over the two substrate binding clefts (Fig. 3c). Meropenem occupies, as expected of a beta-lactam antibiotic, the donor site, further demarcating the adjacent and unoccupied acceptor site. The corresponding volume under the cap in the closed conformation observed is ~810 Å^3^, as calculated by the 3V server^20^ (Supplementary Fig. 4).

### YcbB L,D-transpeptidase mechanism

From the structure of meropenem acylated YcbB, we propose a catalytic mechanism of the L,D-transpeptidase activity of YcbB, which despite the architectural differences is in keeping to what has been proposed for other L,D-transpeptidases previously^21^ (Supplementary Fig. 5). In the proposed mechanism, the conserved Cys528 carries out a nucleophilic attack on the penultimate residue, *meso*-DAP, of a tetrapeptide on the donor PG strand, resulting in the release of the terminal D-Ala. This acyl-enzyme intermediate is subsequently deacylated via nucleophilic attack of a side chain amino on the meso-DAP on an adjacent acceptor PG strand. Conserved His509 of the catalytic dyad is seen to be located in close proximity to the catalytic Cys528 (distance Nτ to acylated Cys S of 4.2 Å); we suggest the hydrogen bond distance and angle between base and nucleophile are presumably optimized only in the apo form of the enzyme due to steric restraints induced by the covalently bound substrate in the subsequent acyl intermediate as captured here. Indeed several structures of acylated and apo forms of PBPs support this with high resolution structures showing the hydroxyl group of the catalytic serine nucleophile swings away from the conjugate base upon acylation^22^. As well, we observe this in our apo molecular dynamics simulations, where the cysteine swings toward His509 within 10 ns of simulation to a final distance of 3.1 Å between the His Nτ to apo Cys S. The backbone carbonyl of the adjacent Asp510 is seen to be located near the Nπ of His509 (2.7 Å), forming a stabilising hydrogen bond and potentially modulating the pK_a_ of the His509 to improve its role as a general base. His509 is seen to be positioned both proximal to not only the catalytic cysteine, but as well the acceptor site of the catalytic cleft (Fig. 3c, d). This supports the notion that His509, stabilised by Asp510, acts as the general base for the activation of both the acylation and deacylation steps of the L,D-transpeptidase reaction. It has been proposed previously for other L,D-transpeptidases that the catalytic histidine may act as both the general base, as well as to protonate the leaving groups^21^, but from our YcbB-meropenem complex structure we propose that Tyr507 is a more likely candidate for this protonation in the case of YcbB. Tyr507 is more appropriately positioned (Supplementary Fig. 1C), in angle and at 4.2 Å from the leaving group nitrogen, than the catalytic histidine, which is positioned 7.2 Å away and appears unlikely to donate this proton. As well, the nearby electropositive Lys497 (4.5 Å away) and potential hydrogen bond partner Trp425 (3.6 Å away) could serve to reduce the pK_a_ of the Tyr507 and allow for its role in protonation of the leaving group. The role of the Tyr507 at this position would be analogous to the role of the SXN motif serine (Ser139 in PBP5) in PBPs^23^, a role which has been very well characterized. We note that while a tyrosine residue exists at this position in L,D-transpeptidases from a variety of other species, it is not completely conserved at this position in the L,D-transpeptidase motif. The specific nature of these protonation events can perhaps be unravelled in future Michaelis complex structures of L,D-transpeptidases.

### Inhibition of the L,D-transpeptidase mediated resistance pathway

**Fig. 4:**
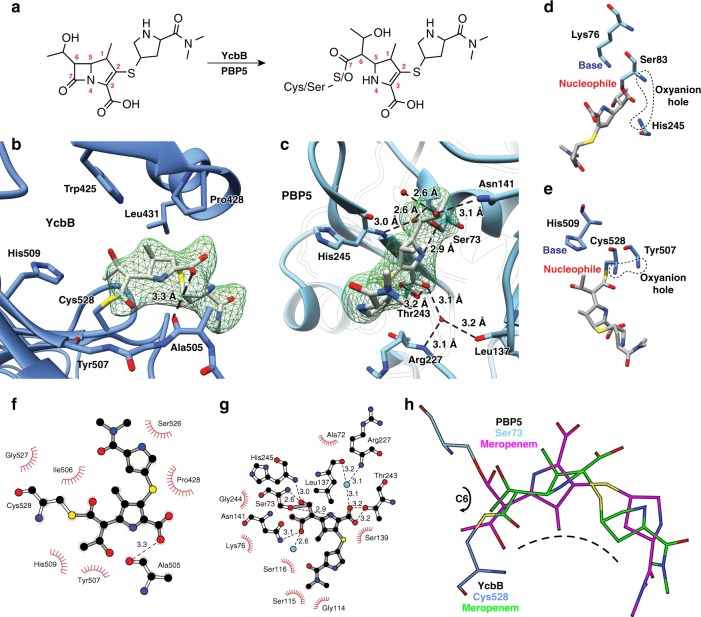
Meropenem inhibition of *E. coli* YcbB and PBP5. **a** Chemical diagram of meropenem pre- and post-acylation by YcbB and PBP5. **b** mF_o_-DF_c_ simulated annealing omit map for the acylated meropenem on Cys528 of YcbB, contoured at 2σ. YcbB is in blue and meropenem in grey. The catalytic dyad (Cys528 and His509), selected residues, and meropenem are coloured by heteroatom. Hydrogen bonding between meropenem and the backbone carbonyl of Ala505 is represented with dashed lines. **c** mF_o_-DF_c_ simulated annealing omit map for the acylated meropenem on Ser73 of PBP5, contoured at 2σ. PBP5 is in blue and meropenem in grey. PBP5 residues involved in the stabilization of meropenem, relevant water molecules, and meropenem are coloured by heteroatom. Hydrogen bonding between meropenem, selected residues, and water is represented with dashed lines. Relative arrangement of nucleophile, general base and oxyanion hole (backbone atoms shown) for PBP5 (**d**) and YcbB (**e**). **f**, **g** LigPlot diagrams of meropenem acyl-enzyme complexes. **f** YcbB-meropenem complex with meropenem in grey and protein residues in white, with atoms coloured by heteroatom. **g** PBP5-meropenem complex coloured as in **f**. **h** Overlay of meropenem-acylated catalytic residues of YcbB (darker blue and green) and PBP5 (lighter blue and pink) showing a similar general curvature of both meropenem molecules, with the notable exception of an approximate 180° rotation about meropenem C6 due to the differing active site architectures of YcbB and PBP5

In order to further probe the inhibition of the YcbB-mediated beta-lactam resistance pathway, meropenem acylation of both the L,D-transpeptidase and D,D-carboxypeptidase activities required was investigated (Fig. 4). The YcbB-meropenem complex map had ordered density defining the active site residues including the thiol ester covalent link of Cys528 and acylated meropenem (Fig. 4b). A 2.2 Å resolution structure of PBP5 acylated with meropenem was also solved with very well ordered density for the covalently bound meropenem (Fig. 4c). Relative to previously published PBP5 structures, we were able to trace additional residues at the C-terminus of PBP5 (387–397; likely afforded by the differential packing with a neighbouring molecule in our unique crystal lattice). Regardless, the conserved binding mode of the ligands between the PBP5-meropenem structure and the PBP5-imipenem structure (PDB ID 3MZF^24^) indicates similar modes of enzyme acylation and binding. On the other hand, despite the similarity in the PG substrates for the YcbB L,D-transpeptidase and PBP5 D,D-carboxypeptidase, we observe the binding and stabilisation of meropenem between the two enzymes is quite distinct.

Meropenem binds PBP5 in a typical PBP fashion using the conserved SXXK, SXN, and KTG active site sequence motifs common to this broad class of activated serine carboxypeptidases. The meropenem is acylated at C7 by the Ser73 side chain hydroxyl of the SXXK motif (the proximal Lys76 of the motif playing the general base role) and stabilised through an extensive hydrogen bond and electrostatic network (Fig. 4c, g). The carbonyl oxygen on C7 of meropenem occupies the enzyme oxyanion hole comprised of main chain nitrogens at position 73 and 245, forming strong hydrogen bonds that polarize and stabilize the carbonyl during acylation and formation of oxycarbanion intermediates (Fig. 4d). The side chain hydroxyl of Ser 139 of the SXN motif resides adjacent to the leaving group nitrogen of meropenem, the likely source for protonation of that moiety to promote acylation. The Asn141 side chain amide of the SXN motif hydrogen bonds with the 1-hydroxyethyl group on C6 of meropenem, while the Thr243 side chain hydroxyl of the KTG motif forms bidentate hydrogen bonds to the carboxylic acid group of meropenem (C3). The latter is further stabilized by longer range electrostatic interactions with the side chain amido of Lys242 of the motif as well as the guanidinium group of Arg277. As well there are typical hydrogen bonds formed, backbone amide of His245 and water-mediated interactions with the backbone amide of Arg227 and the backbone carbonyl of Leu137.

In contrast, meropenem binding of YcbB is void of this extensive network of hydrogen bonds, instead consisting of acylation at C7 by the catalytic Cys528 and a few key hydrogen bonds (Fig. 4b, f). The carbonyl oxygen on C7 of acylated meropenem protrudes into an oxyanion hole consisting of, analogous to the PBPs, the amide nitrogen of the nucleophile, here Cy528. However, the second commonly observed main chain nitrogen amide of the PBPs is not present in YcbB, instead a close interaction to the carbonyl oxygen at Ala 505 with substrate is observed. A further markedly distinct feature in the meropenem complex is the minimal non-covalent interactions of the C3 carboxylate of meropenem with enzyme. One hydrogen bond to the backbone carbonyl of Ala505 is the primary interaction, replacing the typical electrostatic interactions observed for this ubiquitous functional group of beta-lactam antibiotics. Despite the relative proximity of electropositive groups such as Arg407, Arg433, Lys434, and Lys497, the proline rich insertion centered at Pro428 appears to provide a structural ridge that prevents significant interaction. This ridge perhaps contributes to the molecular basis for generally poor acylation of YcbB by classic beta-lactam antibiotics compared to classical PBPs, where the electrostatic positioning of the substrate carboxylate by conserved electropositive side chains is a critical, reoccurring theme. At the same time hydrophobic interactions between enzyme and substrate appear more prominent, with, for example, Trp425, Pro428 and Leu431 forming an explicit apolar pocket for binding of the C1 methyl of meropenem. The general lack of complex hydrogen bonding and increased hydrophobic interactions is characteristic of L,D-transpeptidase acyl-enzyme complexes with carbapenem inhibitors and is seen in meropenem acylation of Ldt_Mt2_ (PDB ID 4QR7^18^). Pre-acylation, there could be additional stabilisation of meropenem from residues of both the active site and the capping loop that allow for proper positioning of the drug for acylation by the catalytic cysteine.

Despite the similarities in the activities of PBP5 and YcbB and therefore some of the common attributes to the inhibition of these enzymes by meropenem, there are major differences in the overall stabilization of these drugs post-acylation. It is likely that PBPs were the target of the evolution of this class of antibiotics, originally in *Streptomyces cattleya* and in subsequent drug development^25^. This is exemplified by structural properties of the inhibition of these enzymes, with the generally more stabilized meropenem of the acylated PBP5 proving a better inhibitor. As we continue to unravel the importance of L,D-transpeptidase mediated beta-lactam resistance, it will become increasingly relevant to revisit this important class of antibiotic and, using structure guided design, improve upon the binding and inhibition of L,D-transpeptidases by carbapenems.

### In vivo assay of YcbB mediated beta-lactam resistance

**Fig. 5:**
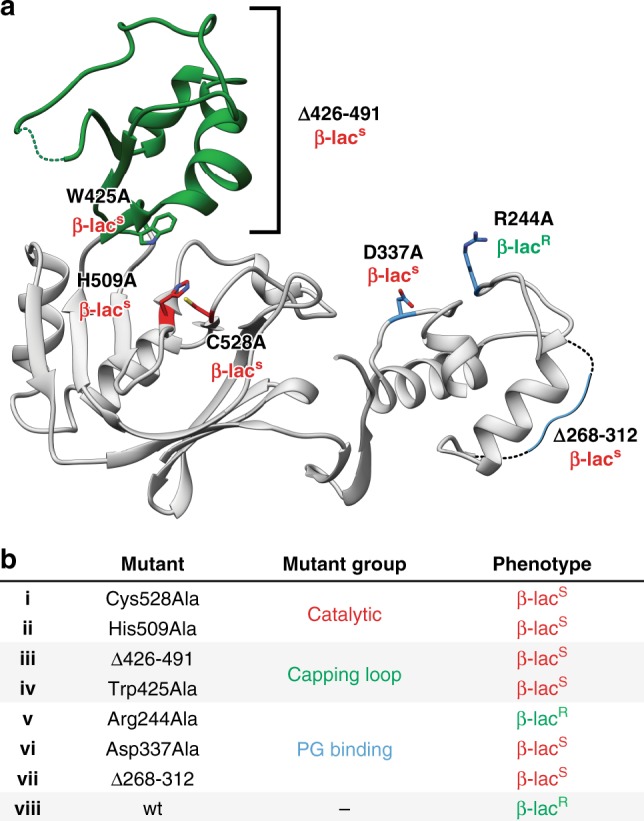
In vivo assay of YcbB mediated beta-lactam resistance. **a** Various replacements and deletions of the catalytic residues (in red), capping loop region (in green) and PG binding domain (in blue), mapped onto the catalytic and PG domains of YcbB. Replacements retaining YcbB mediated beta-lactam (ampicillin and ceftriaxone) resistance are denoted as β-lac^R^, while replacements and deletions resulting in beta-lactam (ampicillin and ceftriaxone) susceptibility are denoted as β-lac^S^. **b** Replacements and deletions of pTK2(*ycbB*) (i–viii) are listed with their resulting phenotype

In order to correlate the sequence and structural aspects of *E. coli* YcbB to its role in antibiotic resistance, antibiograms using a beta-lactam disk diffusion assay were performed (Fig. 5). The antibiotics ampicillin and ceftriaxone were chosen for use in these assays, as they are well characterized, non-carbapenem, beta-lactam antibiotics that provide insight into the general trends in beta-lactam resistance apart from carbapenems. Unsurprisingly, replacements of both the catalytic cysteine and histidine resulted in a complete loss of the beta-lactam resistance (β-lac^R^) phenotype seen in the assay with wild type YcbB.

Of greater interest was the contribution of the capping loop to antibiotic resistance. To this end, the assay was performed with a truncated YcbB containing a YkuD-like capping stub (*ycbB* Δ426–491), as well as with a Trp425 Ala replacement designed to interfere with the significant hydrophobic interactions the indole side chain was observed to mediate with meropenem in our complex structure. Both of these replacements resulted in an beta-lactam sensitivity (β-lac^S^) phenotype. This further validates the proposition that the capping loop, despite its inherent flexibility, plays a crucial role in facilitating YcbB mediated L,D-transpeptidation.

Replacements of the catalytic domain and capping sub-domain provide insight into the requirement for functional L,D-transpeptidase activity in antibacterial resistance, yet an understanding of the role of the PG domain in this resistance and the activity of L,D-transpeptidases in general was poorly understood. While many L,D-transpeptidase enzymes have PG domains of one type or another, these domains have yet to be specifically linked to their role in LD-transpeptidation in vivo. Replacements of both the presumed PG binding residues of the YcbB PG domain (Asp337 and Arg244), as well as a truncation to the extended loop (ycbB Δ268–312) unique to the YcbB PG domain (in comparison to homologous PG domains in other enzymes), were tested using the in vivo beta-lactam resistance assay. Interestingly, only the Asp337Ala replacement and Δ268–312 deletion resulted in beta-lactam sensitivity. The Arg244Ala replacement resulted in a continued beta-lactam resistance phenotype. We propose that this is likely due to a greater contributing role of Asp337 in the binding of PG in comparison to Arg244. Asp337 is positioned more closely to the cleft between the catalytic and PG domains (Fig. 5a), where the acceptor PG strand would likely lie in order to have access to the acceptor site of the catalytic domain. In contrast, Arg244 is positioned further away from the cleft and less likely to be forming as strong an interaction with the acceptor PG strand (Fig. 5a). Regardless of the contribution of Arg244 in PG binding, this is the first evidence of how an accessory PG binding domain can have implications on L,D-transpeptidase function in an in vivo setting. As the extended loop of the PG domain is on the opposite face from residues proposed to be involved in binding the acceptor PG strand, it is possibly mediating additional interactions with the existing PG sacculus that are important in the context of the greater YcbB mediated resistance pathway. Overall, this clearly shows the importance of the accessory PG binding domains in L,D-transpeptidase function in vivo.

### Interaction of YcbB with PBP1b and PBP5

Ycbb, PBP5 and PBP1b are known to contribute sequentially in the D,D-transpeptidase bypass pathway in *E. coli*^5^. It was of interest, therefore, to determine whether YcbB interacts directly with these PBPs that provide substrate. To do this assessment, YcbB-PBP1b and YcbB-PBP5 interactions were assayed using microscale thermophoresis (MST). Styrene-maleic acid co-polymer solubilised PBP1b was used to mitigate protein aggregation issues in the capillary that were encountered with detergent-solubilised PBP1b. The YcbB-PBP1b interaction was seen to have a *K*_d_ of 250 ± 30 nM (Supplementary Fig. 6A). The YcbB-PBP5 interaction was seen to be somewhat weaker, with the MST data suggesting at most a *K*_d_ in the low micromolar range (~2 µM) (Supplementary Fig. 6B). These affinities are consistent with the generally-accepted understanding regarding the dynamic nature of PG biosynthetic complexes in response to environmental challenges or the particular stage in the life cycle of the bacterium^26^. Due to the relatively apolar nature of the scaffolding domain of YcbB (Fig. 1) we postulate that these interactions with PBP1b and PBP5 primarily occur through interactions with apolar regions of PBP1b and PBP5. Both PBP1b and PBP5 are seen to exhibit similar electrostatic surface charge characteristics to YcbB, with localized electropositive surface regions about their active sites and more apolarity to their other surface regions (Supplementary Fig. 7). The association of YcbB to the apolar hinge region of PBP5 and the apolar regions of PBP1b’s glycosyltransferase domain would localise YcbB to receive the proper substrates from PBP1b and PBP5 to successfully mediate beta-lactam resistance.

### Implications on YcbB mediated beta-lactam resistance

**Fig. 6:**
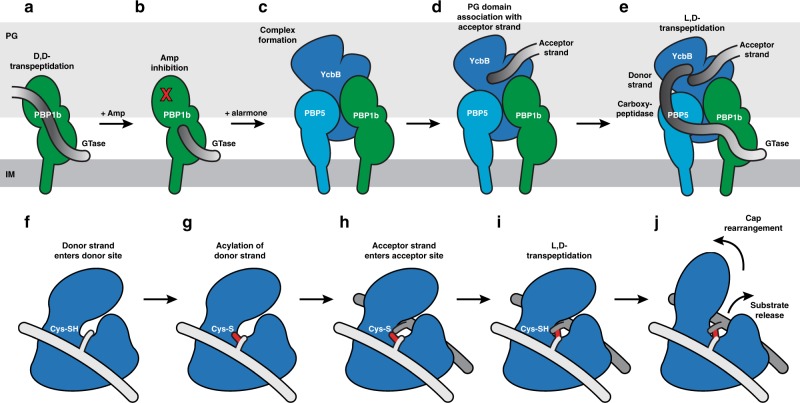
Schematic representation of YcbB mediated beta-lactam resistance. **a**–**e** Assembly and function of the YcbB mediated beta-lactam resistance pathway. **a** PBP1b function in the absence of beta-lactam antibiotics. PBP1b acts as both glycosyltransferase and D,D-transpeptidase. **b** PBP1b function in the presence of beta-lactam antibiotics, such as ampicillin. PBP1b can maintain glycosyltransferase activity, but D,D-transpeptidation is inhibited. **c** Beta-lactam resistance complex formation upon production of alarmone. YcbB likely interacts with PBP1b and PBP5 through its putative interaction/helical domain. **d** YcbB is likely additionally associated and oriented through its PG binding domain interacting with the acceptor strand of the existing PG sacculus. **e** The resistance complex can successfully rescue crosslinking function. PBP1b polymerizes PG strands with glycosyltransferase activity, PBP5 removed a terminal D-alanine with its carboxypeptidase activity, and YcbB acts on the modified peptides with L,D-transpeptidase activity to crosslink the PG strand into the sacculus. **f**–**j** Function and mechanism of the YcbB catalytic domain during L,D-transpeptidation. **f** Tetrapeptide of the donor PG strand enters the donor site of the catalytic domain. **g** Donor tetrapeptide is acylated at its *meso*-DAP residue by the catalytic cysteine, resulting in the loss of the terminal D-alanine. **h** Adjacent acceptor PG peptide enters the acceptor site of the catalytic domain. **i** The covalent acyl-enzyme is deacylated via nucleophilic attack of the side chain *meso*-DAP on the adjacent acceptor PG strand, forming the L,D-crosslink. **j** Rearrangement of the capping loop/sub-domain from a capped conformation to an open cleft will occur, resulting in the release of the crosslinked substrate

The structures reported here further develop our understanding of YcbB mediated beta-lactam resistance and allow us to expand upon the previously proposed model of the resistance pathway^5^ (Fig. 6). Under conditions of increased alarmone production, YcbB and PBP5 are likely able to form a transient complex with PBP1b. The soluble protein YcbB is likely able to couple to the two monotopic membrane proteins, PBP1b and PBP5, to interact directly through its putative interaction/helical domain. As well, YcbB is likely assisted in this localisation and oriented in position to crosslink a polymerising PG strand by its PG binding domain mediated interaction with the existing sacculus. This is distinct from the role of the LysM-like PG binding domain of YkuD^27^ and other Ldt enzymes, in which the PG binding domain is oriented adjacent to the donor site of the catalytic domain, as opposed to the acceptor site seen in YcbB (Supplementary Fig. 8). Once the complex has been formed, PBP1b could act to polymerise periplasmicaly oriented lipid II into a growing PG strand. As beta-lactam drugs inhibit the transpeptidase domain of PBP1b, PBP5 could then assist in rescue of PG formation by removing the terminal D-Alanine, thereby providing the substrate for YcbB. This growing strand would orient itself adjacent to the donor site of YcbB, with the tetrapeptide stem entering into the catalytic cleft. In parallel, the PG domain associated acceptor PG strand would enter the acceptor site of the catalytic cleft. YcbB mediated L,D-transpeptidase activity and substrate release would occur involving rearrangement of the substrate capping loop/sub-domain and resulting in the capped substrate cleft (seen in the acyl-enzyme complex structure) to re-orient into an open cleft. This proposed mechanism for this resistance pathway is consistent with the growing understanding in the field that PG biosynthesis depends on a number of dynamic and variable multi-protein complexes^26^ that are regulated spatiotemporally and, as in the case of YcbB mediated beta-lactam resistance, by external stimuli. As we enter into an era of increasing antibiotic resistance, unravelling the intricacies of these non-canonical PG biosynthetic complexes will be of increasing importance as bacteria deploy these flexible pathways to evade antibiotic assault.

## Methods

### Cloning and protein expression

*Escherichia coli* YcbB without its signal peptide (residue 31 onward) was cloned into the expression vector pET41b-GST with a thrombin cleavable, C-terminal 8x His-tag. *E. coli* PBP1b from residue 58 onward was cloned into the expression vector pET41b-GST with a thrombin cleavable, C-terminal 8x His-tag. *E. coli* PBP5 from residue 30 onward was cloned into the expression vector pET41b-GST with a human rhinovirus (HRV) 3C protease cleavable, C-terminal 8x His-tag. Expression constructs were transformed into *E. coli* BL21 (DE3) for expression of YcbB and PBP5. PBP1b expression construct was transformed into *E. coli* C41. Primers for cloning are listed in Supplementary Table 2. Cells were cultured in ZYP-5052 autoinduction media for 4 h at 37 °C followed by overnight protein expression at 25 °C. Cells were pelleted and stored at −80 °C until required.

### Protein purification

For purification of YcbB and PBP5, cell pellets were resuspended in lysis buffer (20 mM Hepes, pH 8.0, 300 mM NaCl, 10% glycerol) and lysed by processing twice with a homogenizer (15 kPa; Avestin). Cellular debris was pelleted by centrifugation at 125,000 × *g* for 1 h. The resultant supernatant was loaded onto a 1/5 mL Ni^2+^-saturated HisTrap HP Sepharose cartridge (GE Lifesciences), washed with 75 mM imidazole in Buffer A (20 mM Hepes, pH 8.0, 300 mM NaCl), and the protein was eluted with 300 mM imidazole in Buffer A. 1 U of HRV 3 C protease or thrombin was added per mg of protein to remove the N-terminal His-tag overnight at 4 °C. Samples were purified further by size exclusion chromatography (SEC) with a Superdex 200 column (GE Lifesciences) equilibrated in Buffer B (20 mM Hepes, pH 8.0, 150 mM NaCl). Fractions containing pure PBP5 or YcbB were pooled and concentrated to 10 to 40 mg/mL. Protein was frozen rapidly in liquid nitrogen and stored at −80 °C until required. For purification of PBP1b, cell pellets were resuspended in lysis buffer (20 mM Hepes, pH 8.0, 300 mM NaCl, 10% glycerol) and lysed by processing twice with a homogenizer (15 kPa; Avestin). Cellular debris was pelleted by centrifugation at 10,000 × *g* for 30 min. The resultant supernatant was centrifuged at 125,000 × *g* for 1 h to pellet membranes. The membranes were solubilised in Buffer A with 1% (w/v) N-dodecyl-D-maltopyranoside (DDM) overnight at 4 °C and loaded onto a 1/5 mL Ni^2+^-saturated HisTrap HP Sepharose cartridge (GE Lifesciences), washed with 75 mM imidazole in Buffer A with 0.016% DDM, and the protein was eluted with 300 mM imidazole. One unit of thrombin was added per mg of protein to remove the N-terminal His-tag overnight at 4 °C. Samples were purified further by SEC with a Superdex 200 column (GE Lifesciences) equilibrated in Buffer B with 0.2% DM. Fractions containing pure PBP1b were pooled and concentrated to 10 to 40 mg/mL. Protein was frozen rapidly in liquid nitrogen and stored at −80 °C until required. SMA-solubilised PBP1b was prepared as above, with the extraction occurring in 2% activated SMA and subsequent buffers having no detergent present.

### Microscale thermophoresis

MST assays were conducted using a Monolith NT.115Pico (NanoTemper). YcbB, PBP5 and PBP1b were fluorescently labelled using Alexa Fluor 647 NHS Ester (Thermo Fisher Scientific). To evaluate the binding of PBP1b to YcbB, increasing concentrations of unlabelled YcbB (1.5 nM–3.1 µM) were used to titrate fluorescently labelled SMA-solubilised PBP1b at a constant concentration (100 nM). To evaluate the binding of PBP5 to YcbB, increasing concentrations of unlabelled PBP5 (120 pM–98 µM) were used to titrate fluorescently labelled YcbB at a constant concentration (15 nM). Experiments were carried out in a Buffer C. Data was analysed using the NanoTemper MO Affinity Analysis software. The PBP1b-YcbB interaction data was analysed using fluorescence values, while the PBP5-YcbB data was analysed using MST. The data was fit to the equation for a binding isotherm with adjustable Hill coefficient.

### X-ray crystallography and structure determination

*E. coli* YcbB protein was crystallized at 20 °C by sitting drop vapour diffusion using 0.2 µL protein solution (30 mg/mL purified protein in Buffer A) and 1 µL of mother liquor (1.44 M lithium sulphate, 0.08 M HEPES pH 7.5, 0.02 M sodium acetate pH 4.6, 0.015 M ammonium sulfate, 4% (w/v) polyethylene glycol (PEG) 2000 monomethyl ether (MME)) with the addition of 1 mM meropenem. The best diffracting YcbB crystal had the addition of a tetrapeptide (L-Ala-D-Glu-*m*DAP-D-Ala) at 1 mM, though no density could be found for this ligand. *E. coli* PBP5 protein was crystallized at 20 °C by sitting drop vapour diffusion using 0.2 µL protein solution (5 mg/mL purified protein in Buffer A) and 1 µL of mother liquor (0.1 M tris pH 7, 7% PEG 400) with the addition of 1 mM meropenem. X-ray diffraction data of YcbB-meropenem-ethylmercury and PBP5 was collected on Canadian Light Source beamline 08B1–1 using crystals flash-frozen in liquid nitrogen with the addition of 20% glycerol to the mother liquor. Data for native YcbB-meropenem was collected on Advanced Light Source beamline 501. For phasing of YcbB, a YcbB-meropenem crystal was soaked in mother liquor supplemented with 1 mM ethylmercury phosphate for 30 s. A single isomorphous replacement (SIR) experiment was carried out using the ethylmercury phosphate soaked crystal. All datasets were processed with XDS^28^, run manually or through Autoprocess^29^. For the SIR dataset, SHARP^30^ was used for phasing, model building was performed by AutoBuild^31^ and refined using Phenix^32^ and Coot^33^. For the native YcbB dataset and the PBP5 dataset, the structures were solved by molecular replacement using Phaser^34^. The SIR YcbB preliminary structure was used as the template for the YcbB structure and PDBID 3MZF was used to phase the PBP5 crystal. These structures were then refined using Phenix^32^ and Coot^33^. The capping loop region of YcbB was refined using density-guided iterative local refinement as implemented in Rosetta^35^. See Supplementary Table 1 for data collection and refinement statistics. See Supplementary Figs. 1 and 9 for example electron density.

### Molecular dynamics simulations

A complete model for the meropenem acyl and apoenzyme forms of YcbB for molecular dynamics simulations was built from our structure of YcbB-meropenem acyl-enzyme complex with missing disordered loop regions built in using ModLoop^36^. This was then solvated and simulation input files were generated using Quick MD Simulator of the online CHARMM-GUI web service^37,38^. Equilibration and production simulations were performed with GROMACS 5.1^39^, using the CHARMM36 all-atom force field^40^ to represent all protein, solvent and ions. Production simulations were run for 10 ns. RMSD calculations were performed using the RMSD Visualizer Tool and HeatMapper of VMD^41^.

### Impact of substitutions in YcbB on beta-lactam resistance

Beta-lactam resistance mediated by bypass the PBPs requires high-level production of the alarmone (p)ppGpp and of YcbB^5^. In this study, we used strain BW25113Δ*relA*::Km^R^ pKT8(*relA’*), which allows the arabinose-inducible production of (p)ppGpp. The strain was transformed with the derivatives of pKT2(*ycbB*) for IPTG-inducible expression of YcbB and pf derivatives with amino acid substitutions. Primers used to clone YcbB with amino acid substitutions are listed in Supplementary Table 2. The phenotype was analyzed using the disk diffusion assay in BHI agar supplemented with 50 µM IPTG, 1% arabinose, or both inducers, as previously described^5^. Disks were loaded with 10 µg of mecillinam, 10 µg of ampicillin, 30 µg of ceftriaxone, 30 µg of tetracycline, 30 µg of chloramphenicol, or 30 µg of kanamycin. Incubation was performed at 37 °C overnight. Resistance to beta-lactams [β-lac^R^] was defined as growth at the contact of disk containing ampicillin and a diameter <17 mm for the inhibition zone around the disk containing ceftriaxone, as previously described^5^. Susceptibility [β-lac^S^] was defined as diameters >20 mm and >35 mm for the inhibition zones around the disks containing ampicillin and ceftriaxone, respectively. We checked that resistance to ampicillin and ceftriaxone was dependent upon the presence of both inducers.

### Reporting Summary

Further information on experimental design is available in the Nature Research Reporting Summary linked to this article.

## Supplementary information

Supplementary Information

Reporting Summary

## Data Availability

Atomic coordinates for the YcbB-meropenem and PBP5-meropenem models have been deposited in the protein data bank with accession codes 6NTW and 6NTZ respectively.
