# Supplementary material for: Structural insight into YcbB-mediated beta-lactam resistance in Escherichia coli
Source: Nat Commun. 2019 Apr 23;10:1849. doi: 10.1038/s41467-019-09507-0 (PMC6478713; doi:10.1038/s41467-019-09507-0)
Supplement: Supplementary file 1 — Supplementary Information [file 41467_2019_9507_MOESM1_ESM.pdf]

Supplementary Information - Structural insight into YcbB-mediated beta-lactam resistance in *Escherichia coli*

Nathanael A. Caveney, Guillermo Caballero, Henri Voedts, Ana. Niciforovic, Liam J. Worrall, Marija Vuckovic, Matthieu Fonvielle, Jean-Emmanuel Hugonnet, Michel Arthur, Natalie C. J. Strynadka

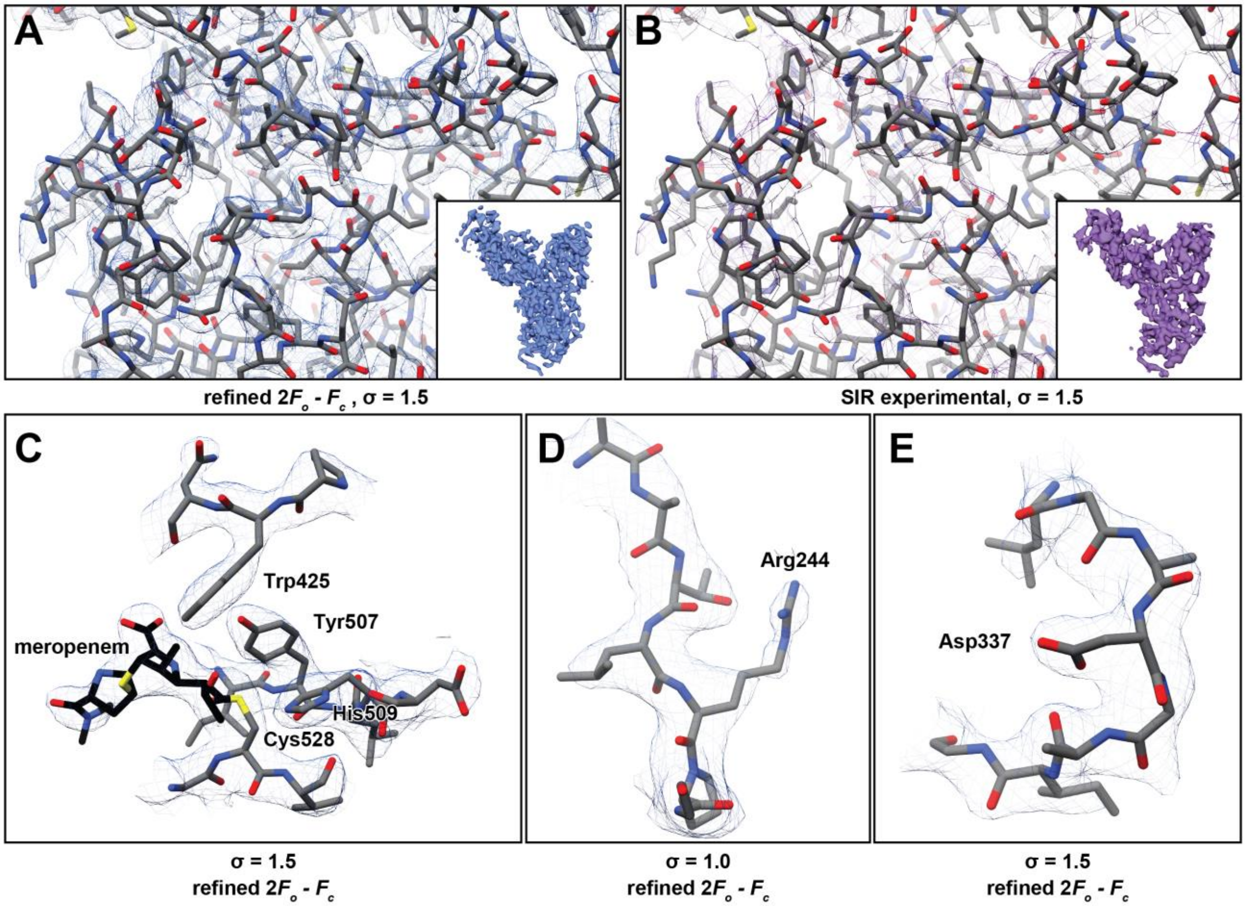

Supplementary Figure 1. Electron density maps. (**A**) Refined 2F_o - F_c map, contoured at σ = 1.5, showing the general map quality after refinement. (**B**) Initial SIR experimental phasing map, contoured at σ = 1.5, at the same position as the refined map in **A**. (**C**) Refined 2F_o - F_c map, contoured at σ = 1.5, about the active site, showing the density for meropenem, Cys528, His509, Tyr507, and Trp425. (**D, E**) Refined 2F_o - F_c maps, contoured at σ = 1.0 and 1.5, about Arg244 and Asp337, respectively.

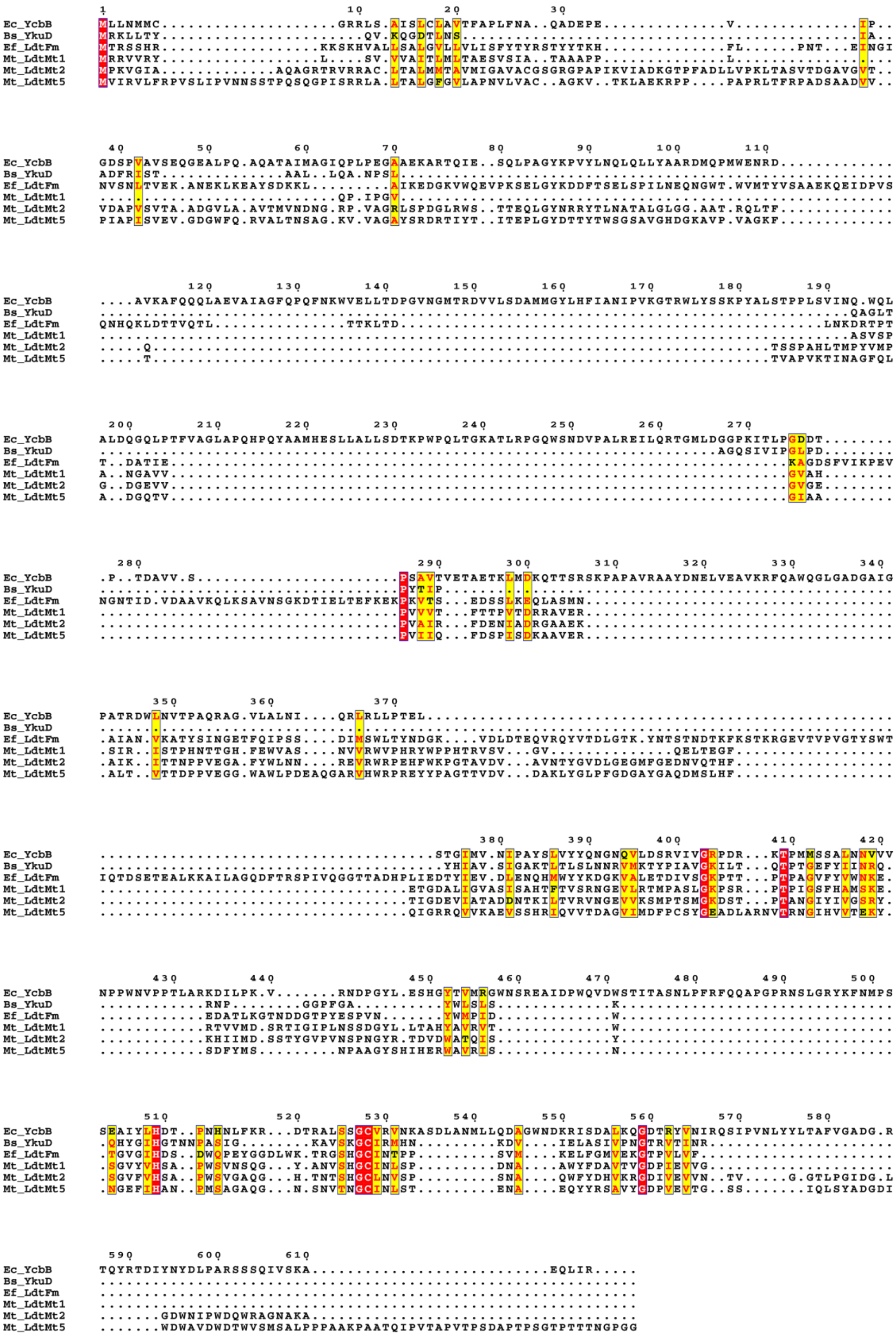

Supplementary Figure 2. L,D-transpeptidase sequence alignments. *E. coli* YcbB aligned with various structurally characterised Gram positive and Mycobacterial L,D-transpeptidases.

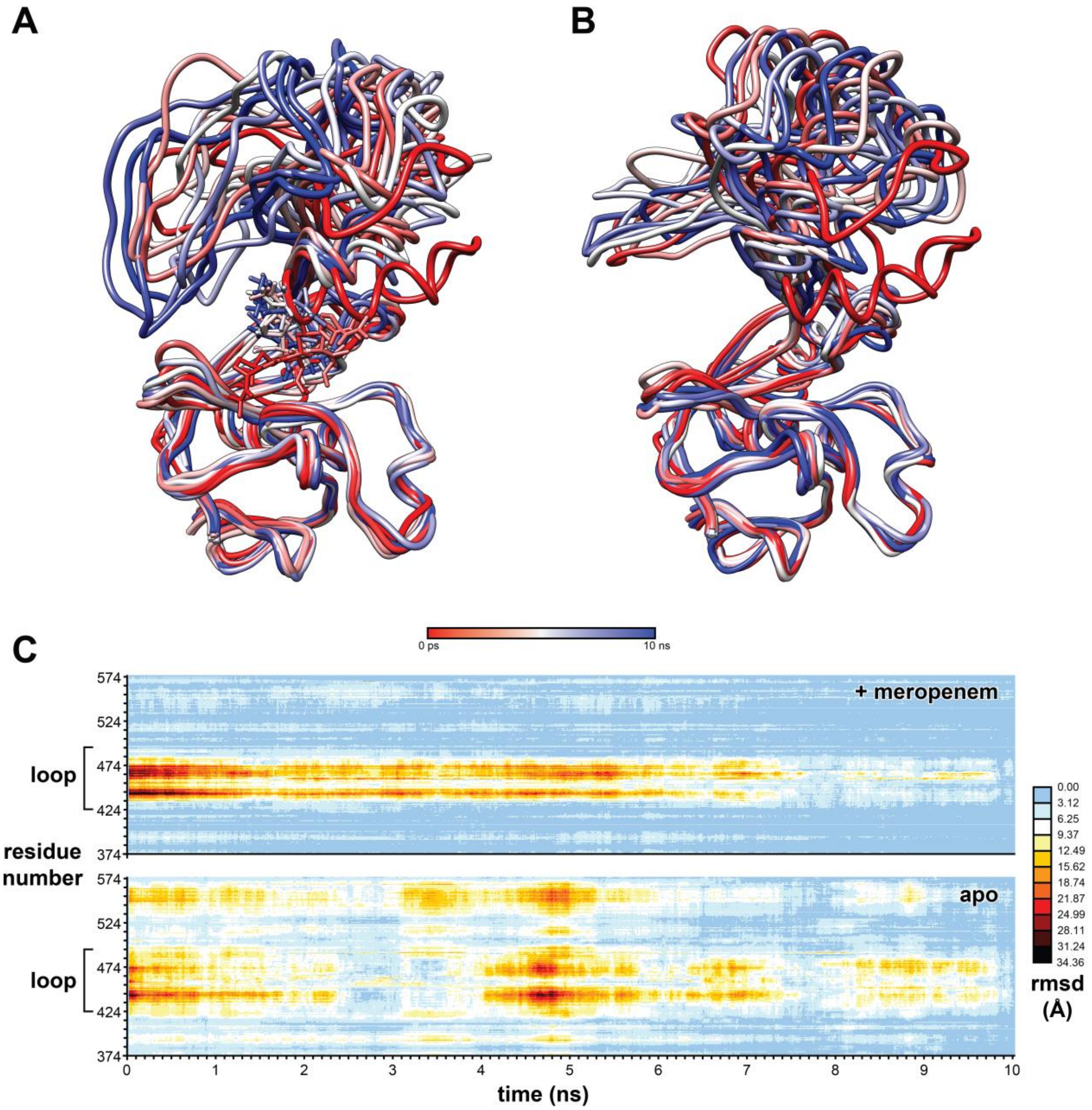

Supplementary Figure 3. Molecular dynamics of *E. coli* YcbB. (**A**) 10 ns simulation of YcbB and meropenem at 1ns increments, catalytic domain show, coloured from red to blue. (**B**) 10 ns simulation of YcbB at 1ns increments, catalytic domain show, coloured from red to blue. (**C**) RMSD of catalytic domain backbone carbons, in angstrom, of YcbB in complex with meropenem (top) and as apoenzyme (bottom).

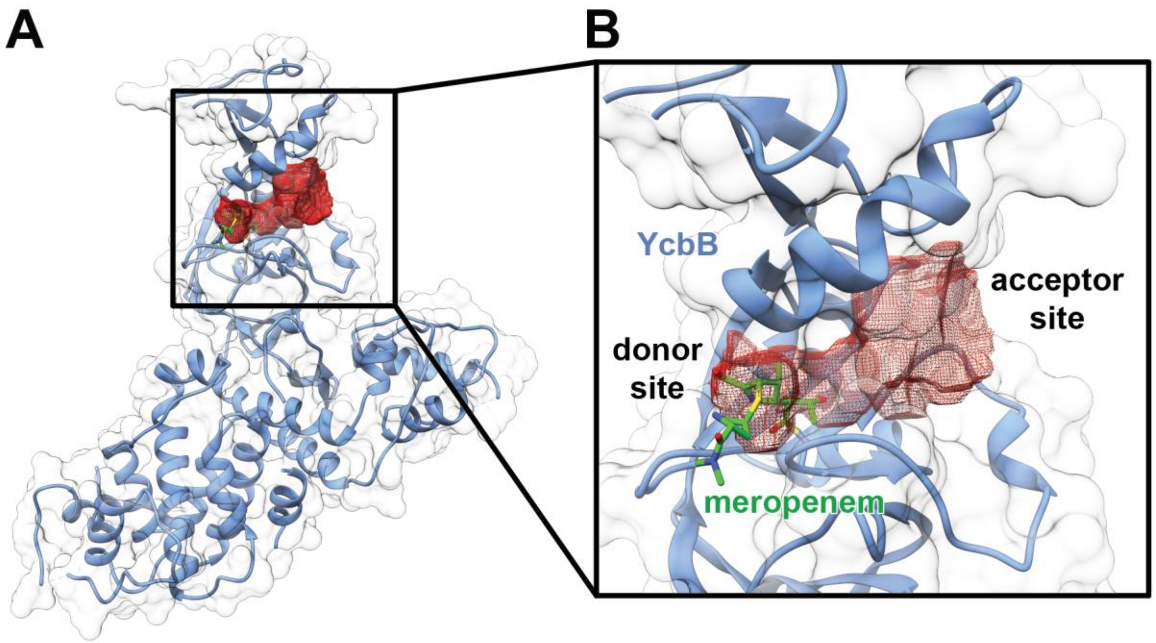

Supplementary Figure 4. Donor and acceptor site volume in YcbB-meropenem acyl-enzyme complex. The 3V calculated volume (811 Å^3) is depicted in red mesh, YcbB in blue, and meropenem in green. Full length depicted in **A** and a view of the catalytic domain in **B**.

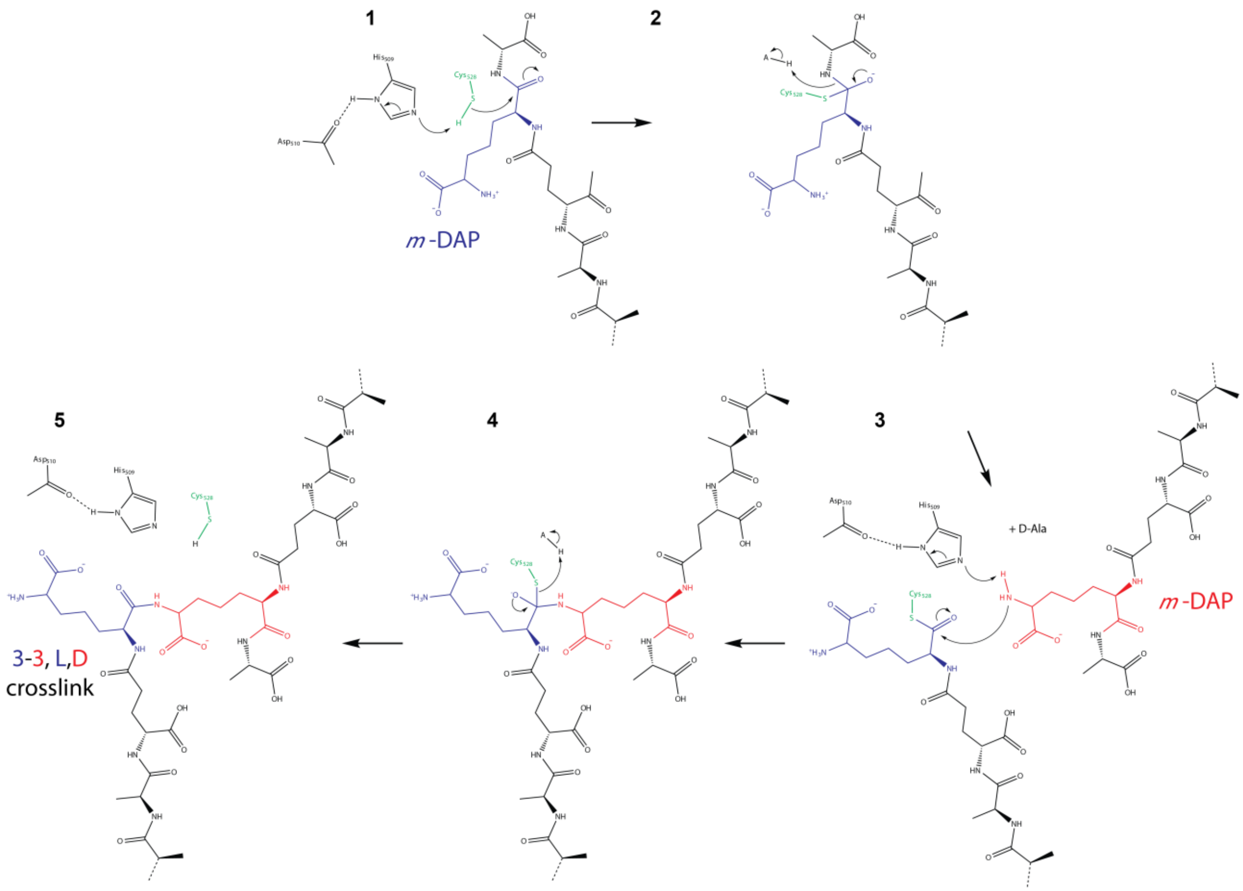

Supplementary Figure 5. Catalytic mechanism of E. coli YcbB L,D-transpeptidase activity. (**1,2**) Activation of Cys528 by His509 and subsequent cysteine mediated acylation of the penultimate residue, *meso*-DAP, of the tetrapeptide on the growing donor PG strand. This results in the release of the terminal D-Ala residue of the tetrapeptide. (**3,4**) Deacylation of the covalent acyl-enzyme via nucleophilic attack of a side chain *meso*-DAP on an adjacent acceptor PG strand. (**5**) The resultant 3-3, L,D crosslink is formed and the enzyme is returned to its starting state.

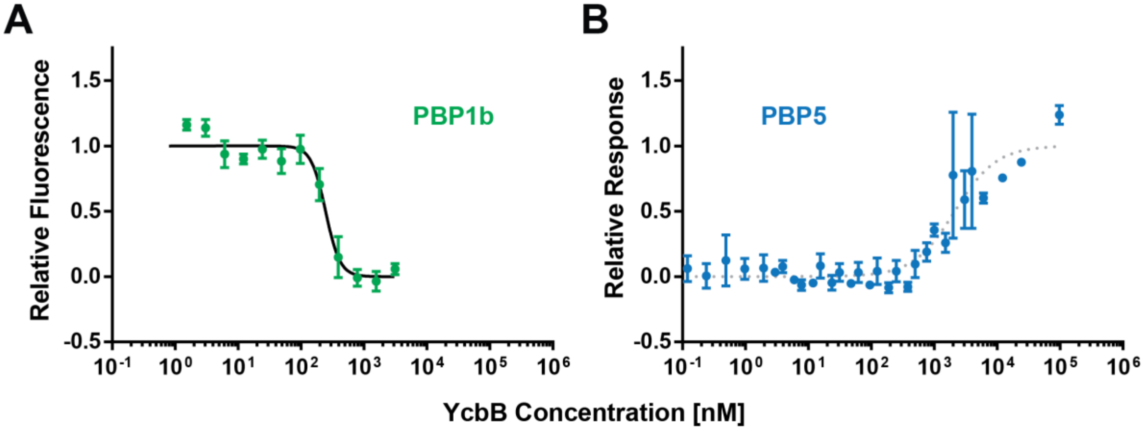

Supplementary Figure 6. *E. coli* YcbB interaction with PBP1b and PBP5. Microscale thermophoresis analysis of PBP1b (**A**) and PBP5 (**B**) interaction with YcbB. (**A**) YcbB-PBP1b interaction was evaluated using relative fluorescence and the data was best fit by a binding isotherm with a K_d of 250 ± 30 nM and a Hill coefficient of 3.8. (**B**) YcbB-PBP5 interaction was evaluated using relative MST response. While it was not possible to work with PBP5 concentrations sufficiently high to observe saturation of all binding sites on YcbB, fitting the data to a binding isotherm yielded K_d and Hill coefficient values of 2.1 ± 0.5 µM and 1.3, respectively. The affinity between YcbB and PBP5 is the estimated maximum affinity for the complex. Measurements for the YcbB-PBP1b interaction were performed in triplicate with two technical replicates of each. Measurements for the YcbB-PBP5 interaction were performed in triplicate. Error bars represent the standard deviation of the measurements in both cases.

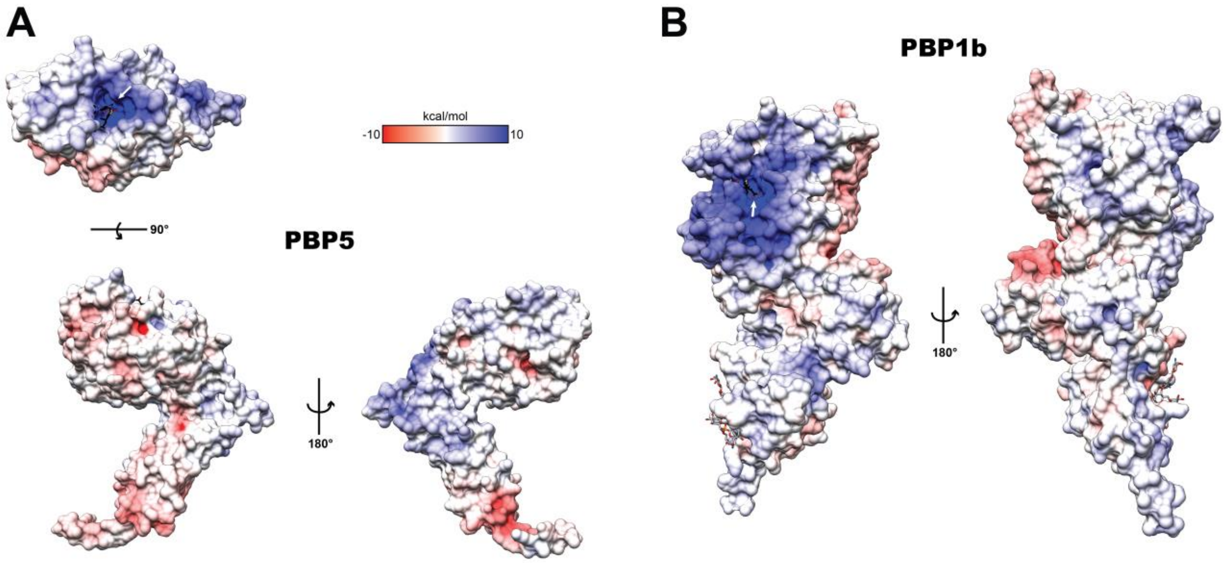

Supplementary Figure 7. Electrostatic surface potential of E. coli PBP5 and PBP1b. (**A**) Electrostatic surface representation of PBP5-meropenem acyl-enzyme complex in three views related by a 90° rotation around the x-axis and a 180° rotation around the y-axis. (**B**) Electrostatic surface representation of PBP1b-CENTA acyl-enzyme complex (PDBID 5HLD[1]) in two views related by a 180° rotation around the y-axis. Electro positive transpeptidase active sites marked with white arrows in both **A** and **B**.

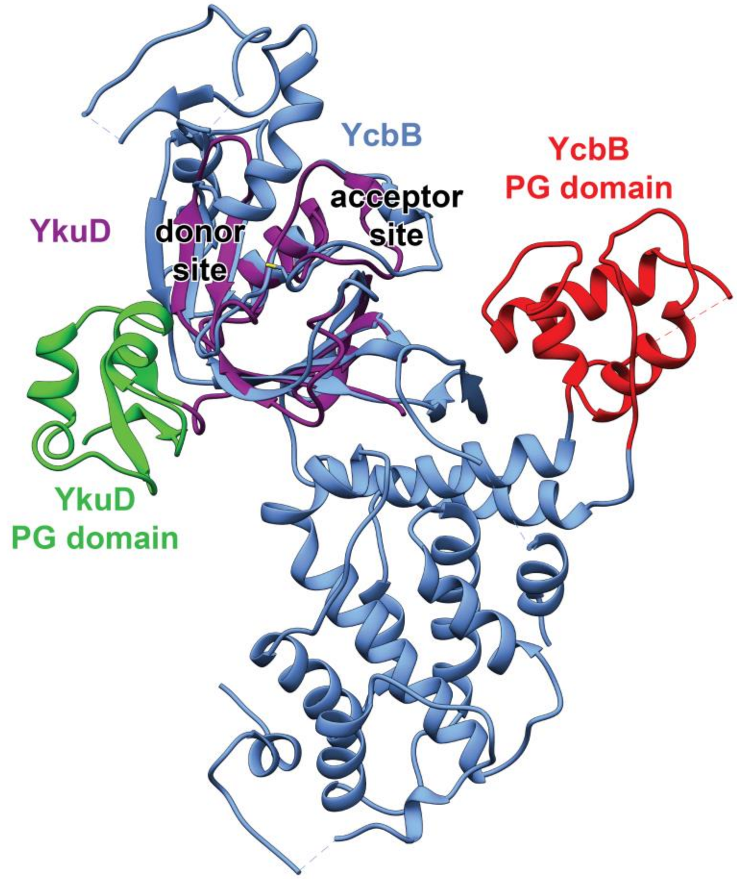

Supplementary Figure 8. PG binding domain orientation of YcbB and YkuD. Overlay of *B. subtilis* YkuD (magenta, 2MTZ[2]) and *E. coli* YcbB (blue), highlighting the position of their respective PG binding domains (green and red, respectively). YkuD PG binding domain has been proposed to interact with the donor strand, while YcbB PG binding domain likely interacts with the acceptor strand of the existing sacculus.

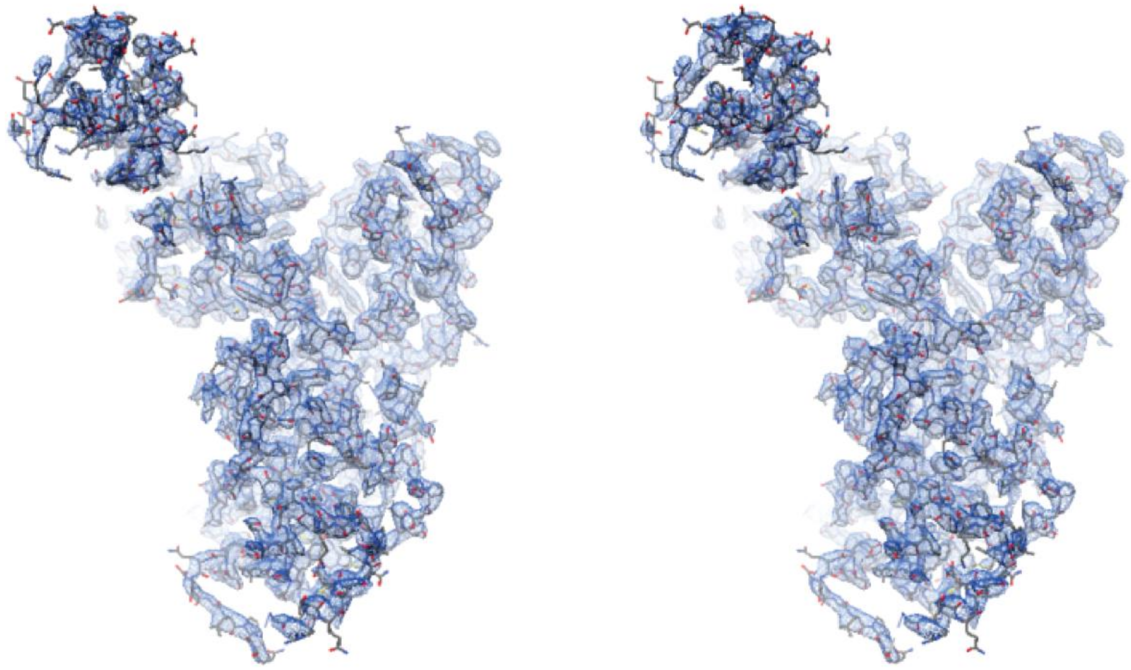

Supplementary Figure 9. Stereo electron density map. Refined $2F_o$ - F_c map, contoured at σ = 1.5, showing the general map quality after refinement.

Supplementary Table 1. Data collection, phasing and refinement statistics

	YcbB Refined	YcbB Native	YcbB Hg	PBP5
**Data collection**				
Space group	P 4_3 2_1 2	P 4_3 2_1 2	P 4_3 2_1 2	C 2
Cell dimensions				
a, *b*, *c* (Å)	126.499, 126.499, 88.8011	126.824, 126.824, 87.903	127.689, 127.689, 87.5560	124.81, 50.8, 80.22
a, b, g (°)	90, 90, 90	90, 90, 90	90, 90, 90	90, 118.688, 90
Resolution (Å)	72.68 - 2.76 (2.859 - 2.76)*	47.92 - 2.19 (up to 3.10 used by SHARP)	47.83 - 2.10	46.08 - 2.2 (2.279 - 2.2)
R_{merge}	0.168 (2.723)	0.075	0.169	0.1767 (1.558)
I / s*I*	9.96 (1.05)	9.2	5	4.86 (0.78)
Completeness (%)	99 (100)	71 (96% up to 2.7A)	89 (100% up to 2.6A)	100 (99)
Redundancy	12.5 (12.8)	6.4	10.7	3.3 (3.2)
R_{cullis}			0.773	
**Refinement**				
Resolution (Å)	2.76			2.2
No. reflections	18942 (1826)			22576 (2204)
R_{work} / R_{free}	25.61 (42.21) / 29.85 (41.93)			21.03 (39.82) / 25.65 (44.21)
No. atoms				
Protein	4066			2942
Ligand/ion	51			26
Water	21			78
B-factors				
Protein	76.8			53.6
Ligand/ion	85.0			70.7
Water	55.7			53.8
R.m.s deviations				
Bond lengths (Å)	0.016			0.006
Bond angles (°)	1.88			0.99

*Values in parentheses are for highest-resolution shell.

Supplementary Table 2. Primers used in this study

Primer	Sequence
**Expression Construct**	
31 → *ycbB* into pET41 - forward	gtttaactttaagaaggagatatacatatggccgatgagcctgaagtaatccctggcgac
31 → *ycbB* into pET41 - reverse	gtcgccagggattacttcaggctcatcggccatatgtatatctccttcttaaagttaaac
**Residue Replacements**	
Cys528Ala – forward	gcattgagctcaggcgctgtacgagtgaataaagcttcc
Cys528Ala – reverse	ggaagctttattcactcgtacagcgcctgagctcaatgc
His509Ala – forward	cagaggccatttatttggctgacacgccgaaccac
His509Ala – reverse	gtggttcggcgtgtcagccaaataaatggcctctg
Δ426-491 – forward	cgtagtggtaaacccgccgtggtcgctggggcgctataaattc
Δ426-491 – reverse	gaatttatagcgccccagcgaccacggcgggtttaccactacg
Trp425Ala – forward	gtagtggtaaacccgccggcgaacgtacctccaactc
Trp425Ala – reverse	gagttggaggtacgttcgccggcgggtttaccactac
Arg244Ala – forward	ggcaaagcaacgttggccccagggcagtggagtaac
Arg244Ala – reverse	gttactccactgccctggggccaacgttgctttgcc
Asp337Ala – forward	caaggattgggggcagctggtgctattggcc
Asp337Ala – reverse	ggccaatagcaccagctgcccccaatccttg
Δ268-312 – forward	gcagcggcatgttggacggggttcgcgccgcctacgataatg
Δ268-312 – reverse	cattatcgtaggcggcgcgaaccccgtccaacatgccgctgc

Supplementary References

1. King, D. T., Wasney, G. A., Nosella, M., Fong, A. & Strynadka, N. C. J. Structural Insights into Inhibition of Escherichia coli Penicillin-binding Protein 1B. *J. Biol. Chem.* **292,** 979–993 (2017).
2. Schanda, P. *et al.* Atomic model of a cell-wall cross-linking enzyme in complex with an intact bacterial peptidoglycan. *J. Am. Chem. Soc.* **136,** 17852–17860 (2014).
